# Challenges in cleaning and disinfection, and environmental monitoring in Swedish slaughterhouses

**DOI:** 10.1186/s13028-025-00838-1

**Published:** 2025-11-13

**Authors:** Madeleine Moazzami, Hedvig Gröndal, Ingrid Hansson, Sofia Boqvist

**Affiliations:** 1 Microbiology Unit, Swedish Food Agency, Uppsala, 751 26 Sweden; 2 Department of Animal Biosciences, Swedish University of Agricultural Sciences, PO Box 7023, Uppsala, 750 07 Sweden

**Keywords:** Cleaning efficacy, Food safety, Post-operational cleaning, Quality control, Slaughterhouse hygiene, Thematic analysis

## Abstract

**Background:**

Cleaning and disinfection (C&D) in slaughterhouses and meat processing facilities is essential to avoid cross-contamination of the meat and thereby prevent food-borne illness and decreased shelf-life of the food product. To determine C&D efficacy, environmental monitoring should be performed. The food business operator must decide which activities to apply in their facility, which can be a challenging task. Ten slaughterhouses, six red meat and four poultry, with associated meat processing facilities participated in this interview study. The animals slaughtered in these slaughterhouses represented approximately 32% and 90% of the annual slaughter in Sweden, respectively. Quality assurance managers of the slaughterhouses were asked 27 questions using digital interviews about their C&D procedures and environmental monitoring. Additionally, the managers could freely elaborate on the difficulties and challenges related to C&D.

**Results:**

Daily C&D was performed in all slaughterhouses and nine hired external cleaning companies. The same type of chemicals were used in all ten slaughterhouses, which primarily included alkaline detergents with or without chlorine for cleaning and chlorine-based agents for disinfection. The most common methods used for monitoring C&D efficacy were the sampling of surfaces by dipslides and ATP-bioluminescence, while one slaughterhouse used swabbing. Only half of the slaughterhouses based thresholds to determine if a surface was sufficiently clean on their own risk-analysis. The remaining slaughterhouses did not provide the information, or the respondent did not know. Quality assurance managers expressed difficulties in determining C&D efficacy, identified several surfaces as difficult to clean and noted reliance on externally provided hygiene thresholds. Four thematic challenges emerged in the thematic analysis: microbial composition on surfaces; efficacy of C&D procedures; competence and management; and production and competitiveness.

**Conclusions:**

Slaughterhouses face notable challenges in C&D, and environmental monitoring, including procedural deficiencies, knowledge gaps, and limited science-based guidelines. Hygiene outcomes are strongly influenced by personnel competence and management support. Limited collaboration between slaughterhouses further impedes the sharing of effective practices. Strengthened partnerships with the scientific community, improved training, risk-based monitoring, and hygienic facility design are essential to enhance C&D standards and reduce microbial contamination risks at slaughterhouses and meat processing facilities.

**Supplementary Information:**

The online version contains supplementary material available at 10.1186/s13028-025-00838-1.

## Background

Adequate cleaning and disinfection (C&D) in slaughterhouses is essential to prevent cross-contamination of the meat with pathogenic and food spoilage microorganisms. According to EU legislation, slaughterhouses must clean and disinfect their facilities and equipment, however, it does not specify how and when the C&D procedure should be performed and monitored [1]. The slaughterhouse hygiene management is governed by standardised operational procedures. These are typically formulated by the facility’s quality assurance unit, approved by relevant regulatory authorities, and subject to validation through internal quality control systems. The procedures are formally documented and maintained in a manner that allows for external auditing, ensuring compliance with national and international standards. General steps of C&D are described in international and national guidelines, but detailed recommendations about e.g. selection of chemical products, concentration, and contact times are generally missing [2, 3]. Consequently, the food business operator (FBO) must decide how to perform the C&D procedure. They often refer to recommendations provided by the manufacturer of the C&D products. While these recommendations are typically based on standardised methods developed in laboratories, they may not fully account for the complex and variable conditions in real-world food processing environment [4, 5].

Commonly used detergents in slaughterhouses and meat processing premises are alkaline compounds with or without chlorine, acids, and enzyme-based chemicals [6–9]. For disinfection, quaternary ammonium compounds (QACs), chlorine-based compounds, acidic agents, and alcohols (i.e. ethanol and isopropyl alcohol) are commonly used [7–10].

The efficacy of the C&D procedures should be evaluated through appropriate environmental monitoring activities [2, 11], including initial visual inspection of surface cleanliness, followed by sampling of surfaces, i.e. tables, conveyor belts, machines, and drains [3, 12]. Various diagnostic methods are used, such as dipslides, ATP-bioluminescence, sampling of rinse water, and swabbing with a sponge or swab [13, 14]. Common hygiene indicators used to assess the C&D efficacy are, for example, total aerobic microorganisms, *Escherichia coli* (*E. coli*), Enterobacterales, yeasts, and moulds [15, 16]. In addition to sampling aimed at quantifying these hygiene indicators, sampling is also conducted to detect pathogenic bacteria such as *Listeria monocytogenes* (*L. monocytogenes*) and *Campylobacter* spp [12, 17]. It may be challenging for FBOs to evaluate the results from bacterial analyses as there are no thresholds included in the current legislation or guidelines. Ideally, thresholds should be based on trends in the number of bacteria on surfaces over time [16, 18].

Several pathogenic bacteria can be transmitted when meat encounters contaminated surfaces, e.g. *Campylobacter*, *Salmonella*, Shiga toxin-producing *E. coli* (STEC), *L. monocytogenes*, and *Yersinia* [17, 19–21]. Moreover, non-pathogenic meat spoilage bacteria being present on meat products may decrease shelf-life and increase food waste [22]. Contamination of bacteria on the meat not only constitutes a public health risk but can also cause economic losses for the FBOs. This is particularly problematic when bacteria can produce biofilm, i.e. *L. monocytogenes* or spoilage bacteria, such as *Acinetobacter* spp., which protects pathogenic and food spoilage bacteria from chemical agents, heat, and desiccation [23, 24]. Once biofilm-producing bacteria become established within a facility, they are difficult to eliminate using common C&D routines [25].

The selection of appropriate C&D activities is crucial in food establishments, notably in slaughterhouses as the surfaces become heavily contaminated on a daily basis. In Swedish slaughterhouses, the quality assurance managers are typically responsible for selecting C&D procedures and monitoring activities, which is a challenging task because limited research studies elaborate on this topic. The aims of this study were to explore how the daily post-operational C&D procedures and environmental monitoring are implemented in the largest slaughterhouses and their associated meat processing facilities in Sweden, and subsequently identify key challenges related to implementation of C&D procedures.

## Methods

### Selection of slaughterhouses

This descriptive study was performed using structured interviews with quality assurance managers at Swedish slaughterhouses slaughtering poultry, cattle, swine, or sheep with associated meat processing facilities. A list consisting of all red meat slaughterhouses was obtained from the Swedish Food Agency, and a corresponding list of the largest poultry slaughterhouses in Sweden was provided by the Swedish Meat Poultry Association. These lists included 21 red meat slaughterhouses and four poultry slaughterhouses. The selection criteria were that there was daily slaughter and that the slaughterhouses were subjected to daily official controls by official veterinarians and auxiliaries. The controls included inspections of the animals before and after slaughter, animal welfare, hygiene routines, animal waste, and Hazard Analysis and Critical Control Points (HACCP). The quality assurance managers of 25 slaughterhouses were contacted through either email or telephone, were informed about the study, and asked if they would be willing to participate anonymously. They were also informed that they could withdraw from the study at any point. Seven slaughterhouses either did not respond or answered that they were interested in participating but later, beyond the study period and were therefore excluded. Eight slaughterhouses declined to participate, with the most frequently stated reason being lack of time. A total of ten slaughterhouses (six predominantly slaughtering cattle and/or pigs and four slaughtering poultry) consented to participate in the study.

### Data collection and analyses

The interviews were conducted digitally (Zoom Video Communications, Inc.), using a questionnaire comprised of 27 questions (see Additional file 1) targeting quality assurance managers and cleaning managers. The questionnaire was developed by the research group, with input from a reference group that included representatives from the Swedish Meat Poultry Association and the Swedish Food Agency. The questions focused on C&D procedures carried out by the slaughterhouses, including the main C&D products being used, and environmental monitoring activities conducted to evaluate C&D efficacy, with the focus on surface samplings. Each interview was expected to take approximately 1.5 h. The quality assurance managers were invited to complement with complementary information by e-mail after the interviews. The interviews were conducted by the first author and the responses were recorded in a Word document during the interview. Following the interviews, the data were reviewed for consistency and clarity. In instances of missing or unclear information, interviewees were contacted for clarification.

The results are presented in two sections, the first section regarding C&D procedures and environmental monitoring are presented descriptively. The second section, “Themes on difficulties and challenges related to cleaning and disinfection” was based on a thematic analysis. Thematic analysis was used to identify and interpret patterns and themes in the interview data. The process followed a structured and sequential approach, and is described as a systematic thematic analysis, which is an approach commonly used within qualitative research, including health-related research [26, 27]. The four steps applied in the thematic analysis were (1) transcription, familiarisation with the data, and selection of quotations; (2) identification of keywords; (3) coding of the data; and (4) theme development. Key words were identified in the quotations, and common codes were generated based on shared characteristics by the first author. These codes were then organised into preliminary themes, which were reviewed and refined, by the entire research team, to ensure they accurately represented the data. This approach enabled a detailed interpretation of how quality assurance staff at slaughterhouses perceive challenges and routines related to C&D.

## Results

### Included slaughterhouses

All four invited poultry slaughterhouses agreed to participate in the study. However, only ten (29%) of the red meat slaughterhouses participated. The most stated reason for not participating was lack of time, and it was mainly the slaughterhouses with the lowest production volumes that declined. Four of the included red meat slaughterhouses slaughtered between 50 and 500 animals/day, and two between 1,500 and 2,000 animals/day (Table 1). Together, these slaughterhouses accounted for 32% of the total number of slaughtered cattle, pigs, sheep, and horses annually in Sweden. The four included poultry slaughterhouses slaughtered between 50,000 and 250,000 animals/day, representing at least 90% of all chickens slaughtered annually in Sweden [28].

**Table 1:** Data from interviews with the largest slaughterhouses in Sweden about their environmental monitoring activities

Slaughter-house	Species	No. slaughtered animals/day	Swabbing for total aerobic bacteria	Dipslides	ATP-tests	Listeria swabs	Origin thresholds clean surfaces	Difficult to clean surfaces
A	Cattle, horse	50–150	na	Once/month	Once/month	Yes (frequency nd)	Supplier of sampling materials	Hooks, platforms, saws, organ table
B	Cattle, swine	50–150	na	na	Once/month	Once/year	nd	Saws, cutting machine, conveyor belts
C	Cattle, swine	1,500–2,000	Na	Once/day	Once/day	4 times/year	nd	Inside machines, dehairing scrapers, singeing machine, organ table
D	Cattle, swine, sheep	1,500–2,000	na	Once/week	na	Once/month	Laboratories	Dehairing scrapers, singeing machine
E	Cattle	50–150	na	2 times/year	Once/month	3 times/year	Laboratories	Saws, knives, organ table
**F**	Cattle, swine, sheep	150–500	na	Once/week	na	Once/week	nd	Platforms, dehairing scrapers, conveyor belts
G	Poultry	50,000–100,000	na	Once/week	na	4 times/year	Supplier of sampling materials	Drills, injectors, knives, pipes, vacuum systems
H	Poultry	100,000–150,000	4 times/year	Once/week	Once/week	Yes (frequency nd)	nd	Inside machines
**I**	Poultry	50,000–100,000	na	na	Once/month	na	Supplier of sampling materials	Cutting facility, transport crates, chilling rooms
J	Poultry	150,000–250,000	na	4 times/week	4 times/week	Once/day	nd	Conveyor belts, displays

*na* not applicable, the slaughterhouse did not use that sampling method, *nd* not determined, the slaughterhouse did not provide the information, or the respondent did not know

### Cleaning and disinfection procedures

In all slaughterhouses, post-operational C&D were performed once daily after slaughter and meat processing were completed, independently of the number of working shifts. All but one poultry slaughterhouse hired an external company for C&D (Table 2). In this particular slaughterhouse C&D were performed by staff members exclusively engaged in C&D activities. All slaughterhouses used alkaline detergents with or without chlorine at low concentrations (2–5%), applied with a low-pressure system.

**Table 2:** Data from interviews with the largest slaughterhouses in Sweden about their cleaning and disinfection procedures

Slaughter-house	Cleaning							Disinfection				
	Species	Operatives	Detergents	Conc (%)	Contact time (min)	Water temp	Application method	Disinfectants	Conc (%)	Contact time (min)	Water temp (°C)	Application method
A	Cattle, horse	External	Alkaline with/without chlorine, acids	3–5	20	20	Foam, low-pressure	Chlorine based	nd	20	20	Foam, low-pressure
B	Cattle, swine	External	Alkaline with/without chlorine, acids	5	15	40–45	Foam, low-pressure	Chlorine based	0.1	15	40–45	Foam, low-pressure
C	Cattle, swine	External	Alkaline with chlorine	4	15–20	nd	Foam, low-pressure	Acids, hydrogen peroxide	4	nd	nd	Foam, low-pressure
D	Cattle, swine, sheep	External	Alkaline with/without chlorine, acids	3	5–20	50	Foam, low-pressure	Chlorine based	3	5–30	50	Foam, low-pressure
E	Cattle	External	Alkaline with chlorine, acids	3–5	10–15	50	Foam, low-pressure	Chlorine based, acids	3–5	10–15	50	Foam, low-pressure
F	Cattle, swine, sheep	External	Alkaline with chlorine, acids	2–5	15–20	< 35	Foam, low-pressure	Chlorine based, acids	1–3	nd	10–20	Foam, low-pressure
G	Poultry	External	Alkaline with/without chlorine, acids	3–5	15–20	20	Foam, low-pressure	Chlorine based	3–5	15–20	15–18	Foam, low-pressure
H	Poultry	External	Alkaline with/without chlorine, acids	3	15–20	nd	Foam, low-pressure	Chlorine based	3	15–20	nd	Foam, low-pressure
I	Poultry	External	Alkaline with/without chlorine, acids	3–5	15–30	35	Foam, low-pressure	Acids	nd	nd	30–35	Foam, low-pressure
J	Poultry	Internal	Alkaline with/without chlorine, acids	4	15–30	55	Foam, low-pressure	Chlorine based, acids	1–3	15–30	55	Foam, low-pressure

Conc = concentration. nd = not determined, the slaughterhouse did not provide the information, or the respondent did not know. External operatives = external cleaning company. Internal operatives = cleaning personnel hired by the slaughterhouse

Almost all (90%) of the slaughterhouses alternated alkaline with acidic agents during cleaning. The contact time for detergents varied between 10 and 30 min in all slaughterhouses except one. That slaughterhouse followed the manufacturer’s recommendation of a shorter contact time (5 min). The temperature of the water used for cleaning ranged from 20 to 55 °C. Most (80%) of the slaughterhouses used chlorine-based agents in the disinfection process, which were applied using low-pressure systems. There was a larger variation in the disinfectants used compared with the detergents, which could explain the wider variation in concentration (0.1–5.1%) and water temperature (10–55 °C). Within all slaughterhouses, the contact time was the same for disinfectants as for detergents. None of the slaughterhouses used QACs and the majority of managers mentioned that surfaces were not always dry in the morning prior to the start of production.

### Environmental monitoring

There was a considerable variation in the number of animals slaughtered at each slaughterhouse (Table 1). Visual inspection of hygiene status was carried out following cleaning by staff from the cleaning company. In addition, visual inspection was also performed by slaughterhouse personnel in the morning prior to the commencement of production.

To monitor environmental cleanliness after C&D, only one slaughterhouse used swabbing and analyses of total aerobic bacteria. The majority of the slaughterhouses regularly used dipslides (80%) and ATP-bioluminescence (70%), and half of the slaughterhouses used a combination of these two methods. Only half of the slaughterhouses applied bacterial thresholds based on risk analysis, while the remaining slaughterhouses either did not provide the information, or the respondent did not know (Table 1). Almost all (90%) of the slaughterhouses included monitored *Listeria* spp. using swabs in their floor drains. Half of the slaughterhouses mentioned that they had detected *Listeria* spp. in their drains. Occasionally chlorine tablets were added to the drains to prevent bacterial growth.

Surfaces mentioned as difficult to clean by at least two quality assurance managers included cutting tools, organ tables, machine interiors, conveyor belts, dehairing scrapers, platforms, and a singeing machine (used for burning the carcass surface). Half of the slaughterhouses relied on the thresholds provided by the external laboratories who analysed the samples, or by suppliers or manufacturers of the sampling materials, instead of establishing their own thresholds based on internal risk analysis.

### Themes on difficulties and challenges related to cleaning and disinfection

In the thematic analysis, four key themes were identified that addressed knowledge gaps in microbial composition on surfaces; efficacy of C&D procedures and the role of scientific advice within the C&D context; staff competence and management; and challenges related to production and competitiveness. The themes are presented below.

#### Theme 1: Microbial composition on surfaces

Several quality assurance managers were interested in mapping the in-house microbiota of their premises and some expressed that they were unable to find laboratories that could perform these types of analysis. They wanted to know which bacteria were present on surfaces. One quality assurance manager stated: “*It would be interesting to investigate specific bacteria causing spoilage and how to eliminate them. We would like to avoid building up a house flora*,* including Pseudomonas*.” Other questions that were raised by the managers were: “*Do we take too few samples?*” and “*When we see a trend in elevated monitoring results*,* are we doing the right thing?*”.

#### Theme 2: Efficacy of C&D procedures

Almost half of the quality assurance managers wanted to know if the C&D procedures applied in their facilities were the most efficient. One manager said: “*We want to know if the cleaning and disinfection products and methods we use are the most appropriate or if there are other more efficient methods available.*” Another manager stated: “*We would like to verify the efficacy of our cleaning and disinfection procedures*.” In addition, the quality assurance managers wanted to gain support from scientists and there was a perceived lack of science-based guidelines. One manager stated: “*It would be nice to have guidelines based on science. We would like to get help from experts*.” Another manager remarked: “*What affects the production of biofilm? How much does the surface material influence?*”

#### Theme 3: Competence and management

To assess the performance of the external cleaning companies, certain slaughterhouses demanded that 95% of the surfaces should be acceptably clean, according to their thresholds for cleanliness. Half of the slaughterhouses had changed their cleaning company over the last five years. The reason for this was that they were unsatisfied with the C&D results. Certain slaughterhouses highlighted the importance of the competence of the cleaning personnel and that it can be difficult to find sufficiently skilled personnel. Good leadership and management at the slaughterhouses were also acknowledged as fundamental as it is at this level that resources for C&D procedures and environmental monitoring are decided: “*Leadership and management are important*.” and “*It is hard to find the right management*.” Approximately half of the quality assurance managers had completed university studies within biological sciences. One respondent had a technical background, and another had only received in-house training, entering the role through a family business connection.

#### Theme 4: Production and competitiveness

Several quality assurance managers expressed a lack of communication regarding C&D practices between slaughterhouses which do not belong to the same company. One manager said: “*It would be interesting to know how they clean and disinfect other slaughterhouses. What cleaning and sampling methods do they use and what results and challenges do they have?*” Another manager mentioned: “*It would be nice to get support and ideas on how to do things differently*,* to share knowledge*.” Some managers stated competition as the reason for the lack of communication between slaughterhouses. One manager stated: “*The feathers are squeezed under the conveyor belts*,* and they have to be taken apart regularly in order to clean the belts properly. Hygienic design has been forgotten by the manufacturers*,* they do not have cleaning in mind*,* just production*.”

## Discussion

This study focused on post-operational C&D, which was performed daily at all included slaughterhouses and complemented additional cleaning during operation. All but one of the participating slaughterhouses hired an external company to perform daily post-operational C&D. This is not surprising, as most slaughterhouses reported having insufficient knowledge of the most efficient C&D procedures. The slaughterhouse that performed C&D with its own staff was the poultry slaughterhouse with the highest slaughter volume. It might be that it is difficult for slaughterhouses to have enough capacity and skills to conduct their own C&D. However, it was reported that half of the slaughterhouses had changed their cleaning company over the last five years as they were unsatisfied with the C&D results. This highlights the need for science-based guidelines for those performing C&D at slaughterhouses.

All four invited poultry slaughterhouses agreed to participate in the study. In contrast, there was a high drop out of the invited red meat slaughterhouse (15 out of 25). It was mainly the smaller red meat slaughterhouses that dropped out and the stated reason was lack of time as there was often only one person at these slaughterhouses that worked with hygiene and environmental monitoring. There is no reason to believe that the red meat slaughterhouses that chose not to participate were better or worse compared to the slaughterhouses that were included in the study. None of the slaughterhouses included in the study used QACs, which was surprising as these compounds are widely used in both poultry and red meat slaughterhouses in other countries [7–9, 29]. However, the use of sodium hypochlorite instead of QAC may be favourable, considering that biofilm and planktonic cells of *L. monocytogenes* show higher resistance to QACs [30]. Furthermore, QACs are known to be less efficient against Gram-negative bacteria [31]. All slaughterhouses used low-pressure systems when performing C&D. This is according to standard as high-pressure systems are not recommended in heavily soiled facilities such as slaughterhouses due to the high pressure efficiently spread soil with microorganisms in the surrounding environment [32]. In addition, the concentration of detergents and disinfectants was in the lower range of what was recommended, which is expected as higher concentrations could be hazardous to workers [32].

In the present study, dipslides measuring total aerobic count and ATP-bioluminescence were mainly used for environmental monitoring, separately or in combination. Each sampling method has its advantages and disadvantages. For instance, dipslides are easy to use and require minimum labour, but have a low sensitivity, and have to be incubated for 48 h [14]. ATP-bioluminescence is also easy to use and provides results within seconds, however, it does not only detect the number of bacterial cells as it also measures cells from organic material such as blood cells and fat, which can affect the accuracy of the results [30]. A suggestion could therefore be to validate dipslides and ATP assays using swab samples [14], and to alternate between these two methods, as 50% of the slaughterhouses did in the present study. Only half of the participating slaughterhouses reported using bacterial thresholds informed by their own risk analysis, while the remainder either did not provide this information or were unsure. This lack of clarity indicates that the implementation of risk-based criteria for microbial monitoring may be inconsistent across facilities. Given that the use of thresholds derived from in-house risk analyses is considered best practice for targeted and effective hygiene control, these results highlight a need for improved documentation, awareness, and application of risk-based approaches within the sector.

If thresholds are derived solely from recommendations by external companies, rather than the FBO’s own risk analysis, there may be an increased risk of applying thresholds that are inappropriate for the in-house microbiota, specific surface or point in the production line. There was no specific question on whether the slaughterhouses checked for trends in the results. However, one slaughterhouse mentioned that they did not know what to do when they observed a negative trend in the environmental monitoring results. It is essential that quality assurance managers act upon increased bacterial counts on surfaces after C&D to prevent cross-contamination [11].

Nine out of ten participating slaughterhouses included analyses for *Listeria* spp. in their environmental monitoring, particularly since some of them also produced ready-to-eat (RTE) meat product. This practise aligns with EU legislation, which states that facilities producing RTE products must monitor the production environment for *L. monocytogenes* [12]. However, some of the included slaughterhouses had implemented measures to control *Listeria* spp. also in cases where RTE products were not produced. This reflects a broader awareness of the challenges associated with *L. monocytogenes.*

The quality assurance managers mentioned several surfaces in the slaughterhouses that were difficult to clean. This was supported by other studies showing that, for example, conveyor belts, dehairing equipment, and cutting tools are challenging to clean [9, 33, 34]. For plastic surfaces such as conveyor belts, a plausible explanation for difficulties in cleaning could be the pronounced bacterial adhesion that has been observed on plastic, which often develops scratches on the surfaces over time [35]. Although drains are non-food contact surfaces, they are recognised as important points for environmental sampling due to their role as a reservoir for pathogens, such as *L. monocytogenes* which is known to persist in drains for several months and even years [36, 37]. While splash or aerosol formation during washing can contribute to environmental spread of bacteria [38], the primary rationale for sampling drains lies in their function as sentinel sites for pathogen detection, providing a comprehensive indication of the facility’s overall hygiene status and potential contamination risks.

Another issue that affects microorganisms on surfaces is the high humidity at the slaughterhouse. Most quality assurance managers mentioned that surfaces were not dry in the morning before the start of production. It is imperative that surfaces are provided the opportunity to dry after C&D to avoid growth and survival of microorganisms [39]. To enhance the drying of surfaces, half of the slaughterhouses used forced ventilation after C&D. Forced ventilation is an efficient way to dry surfaces, however it is a costly investment [17].

Several of the quality assurance managers representing the slaughterhouses mentioned that they had insufficient knowledge of which C&D procedures would be the most efficient in their slaughterhouse. These are relevant concerns as it is challenging to compare procedures and determine all the variables to take into consideration. Most commonly, determination of the efficacy of different chemicals against various bacteria are performed in laboratory as opposed to real-life settings [40–42]. Understanding the industry’s perceived challenges related to C&D routines is essential for effectively translating findings from laboratory studies into real-world applications. The comments regarding perceived lack of science-based guidelines are concerning, as they highlight difficulties in knowledge transfer between the scientific community and the food industry [43].

Some quality assurance managers expressed interest in exchanging experiences related to C&D, with assurance managers at other slaughterhouses. These kinds of discussions could be important before implementing a new sampling plan, particularly when establishing thresholds to determine whether a surface is acceptably clean. Bacterial thresholds should be clearly defined and accompanied by appropriate corrective actions if they are not met [12]. Although slaughterhouses may collaborate in certain areas related to production, this might not be the case for C&D. The slaughterhouses, while operating within the same sector, are commercial competitors, which may limit openness and knowledge exchange on topics perceived as closely linked to internal quality control practices. Furthermore, C&D may not be prioritised to the same extent as core production activities, which could contribute to fewer structured discussions or joint initiatives in this area. These factors may explain why the type of dialogue necessary for establishing shared or improved C&D sampling strategies and thresholds was not reported by the respondents.

Thematic analysis was chosen as the analytical approach for the qualitative part of this study to allow for a systematic and transparent interpretation of the interview data [26, 27]. This method enabled the identification and organisation of recurring patterns related to perceived challenges in C&D procedures among quality assurance staff. By using this approach, we were able to capture both individual and shared perspectives across slaughterhouses, thereby facilitating a comprehensive understanding of the data and strengthening the connection between data collected using questionnaire and final conclusions.

## Conclusions

This study explored challenges related to C&D, and environmental monitoring practices in slaughterhouses and meat processing facilities. Although post-operational daily C&D routines were in place across all participating FBOs, the results indicate variations in procedures, chemical usage, and monitoring strategies. Quality assurance managers described uncertainties related to microbial composition on surfaces, the efficacy of C&D protocols, and the availability of science-based guidelines. The competence of cleaning personnel and the level of managerial engagement were frequently mentioned as factors perceived to influence C&D outcomes. Additionally, there was limited exchange of experiences specifically related to C&D reported. Further consideration of training, hygienic design, and risk-based monitoring thresholds may contribute to improved implementation and consistency of C&D practises.

## Supplementary Information

Supplementary Material 1.

## Data Availability

The datasets used and/or analysed during the current study are available from the corresponding author on reasonable request.

## Declarations

### Prior publication

Part of the study is published in Moazzami’s PhD thesis (https://pub.epsilon.slu.se/32048/1/moazzami-m-20231102.pdf).
