# Supplementary material for: Challenges in cleaning and disinfection, and environmental monitoring in Swedish slaughterhouses
Source: Acta Vet Scand. 2025 Nov 13;67:47. doi: 10.1186/s13028-025-00838-1 (PMC12616898; doi:10.1186/s13028-025-00838-1)
Supplement: Supplementary file 1 — Supplementary Material 1. [file 13028_2025_838_MOESM1_ESM.docx]

**Interview questions for quality assurance managers at slaughterhouses with associated meat processing facilities**

*C&D = Cleaning and disinfection

General questions:

1. What is your educational background?

2. What animal species are slaughtered in your facility?

3. How many animals are slaughtered each day?

Cleaning and disinfection procedures:

4. Who performs C&D (external/internal cleaning staff)?

5. Have you changed cleaning company during the past 5 years?

If answer to question 5 is yes:

6. Why have you changed the company?

7. How often is C&D performed?

8. Are surfaces dry when the slaughter/meat processing starts?

Detergents:

9. Which agents are used?

10. At what concentration are they diluted in water?

11. How long is the contact time, *i.e.* how long are the products left to act before they are rinsed away?

12. What is the temperature of the water used for cleaning?

13. How are the agents applied on surfaces?

14. Why did you choose the parameters above (question 9-13)

Disinfectants:

15. Which agents are used?

16. At what concentration are they diluted in water?

17. How long is the contact time, *i.e.* how long are the products left to act before they are rinsed away?

18. What is the temperature of the water used for cleaning?

19. How are the agents applied on surfaces?

20. Why did you choose the parameters above (question 15-19)

21. Is there a rotation between the chemical agents used (Yes/No)?

If answer to question 21 is Yes:

22. Which chemicals do you alternate between?

Monitoring activities:

23. Which sampling methods are used to evaluate C&D efficacy?

24. With what frequency are these sampling methods performed?

25. How did you select the microbial and non-microbial threshold/s for clean surfaces?

26. Which surfaces do you think are the most difficult to clean and should be sampled?

Challenges and difficulties related to C&D:

27. What difficulties and challenges have you experienced in relation to C&D?
